# Large-scale functional expression of WT and truncated human adenosine A_2A_ receptor in *Pichia pastoris *bioreactor cultures

**DOI:** 10.1186/1475-2859-7-28

**Published:** 2008-10-10

**Authors:** Shweta Singh, Adrien Gras, Cédric Fiez-Vandal, Jonathan Ruprecht, Rohini Rana, Magdalena Martinez, Philip G Strange, Renaud Wagner, Bernadette Byrne

**Affiliations:** 1 Membrane Protein Crystallography Group, Division of Molecular Biosciences, Imperial College London, South Kensington, London, SW7 2AZ, UK; 2 Unité Mixte de Recherche 7175 École Supérieure de Biotechnologie de Strasbourg – Centre National de la Recherche Scientifique, Département Récepteurs et Protéines Membranaires, 67412 Illkirch, France; 3 School of Pharmacy, PO Box 228, University of Reading, Whiteknights, Reading RG6 6AJ, UK; 4 Karolinska Institutet, Department of Medical Biochemistry and Biophysics (MBB), Division of Biophysics, Scheeles väg 2, 171 77 Stockholm, Sweden

## Abstract

**Background:**

The large-scale production of G-protein coupled receptors (GPCRs) for functional and structural studies remains a challenge. Recent successes have been made in the expression of a range of GPCRs using *Pichia pastoris *as an expression host. *P. pastoris *has a number of advantages over other expression systems including ability to post-translationally modify expressed proteins, relative low cost for production and ability to grow to very high cell densities. Several previous studies have described the expression of GPCRs in *P. pastoris *using shaker flasks, which allow culturing of small volumes (500 ml) with moderate cell densities (OD600 ~15). The use of bioreactors, which allow straightforward culturing of large volumes, together with optimal control of growth parameters including pH and dissolved oxygen to maximise cell densities and expression of the target receptors, are an attractive alternative. The aim of this study was to compare the levels of expression of the human Adenosine 2A receptor (A_2A_R) in *P. pastoris *under control of a methanol-inducible promoter in both flask and bioreactor cultures.

**Results:**

Bioreactor cultures yielded an approximately five times increase in cell density (OD600 ~75) compared to flask cultures prior to induction and a doubling in functional expression level per mg of membrane protein, representing a significant optimisation. Furthermore, analysis of a C-terminally truncated A_2A_R, terminating at residue V334 yielded the highest levels (200 pmol/mg) so far reported for expression of this receptor in *P. pastoris*. This truncated form of the receptor was also revealed to be resistant to C-terminal degradation in contrast to the WT A_2A_R, and therefore more suitable for further functional and structural studies.

**Conclusion:**

Large-scale expression of the A_2A_R in *P. pastoris *bioreactor cultures results in significant increases in functional expression compared to traditional flask cultures.

## Background

G-protein-coupled receptors (GPCRs) form a large superfamily of cell-surface receptors that mediate cellular responses to a wide range of biologically active molecules including hormones, neurotransmitters and drugs. Indeed, half of all currently available pharmaceuticals act through GPCRs [1,2]. The physiological and pharmacological importance of these proteins makes them key targets for drug discovery programmes. Our understanding of the precise mechanism of action of these important proteins is currently limited by a lack of high-resolution structural data. One limiting factor to structural studies of GPCRs has, until recently, been low expression levels [3]. With the exception of rhodopsin, all GPCRs are expressed at very low levels endogenously, thus requiring the development of recombinant overexpression systems. Careful expression vector design, GPCR codon-optimisation [4] and high throughput approaches used to identify GPCRs with the highest expression levels in different expression systems [5] are among the methods that have been used to produce sufficiently high levels of functional GPCRs suitable for structural studies.

Success has been achieved when using the expression host *Pichia pastoris *for the production of membrane proteins for structural studies, most notably the rat membrane protein K^+ ^channel [6]. *Pichia *has several advantages over other systems for the production of GPCRs. It is easy to manipulate, has high production levels and is relatively inexpensive. In addition, *Pichia *has the ability to glycosylate expressed receptors, albeit in a modified form compared to higher eukaryotes, which is essential for the proper functioning and membrane targeting of many receptors [7-10]. Much effort has been applied to the optimisation of *Pichia *expression systems specifically for GPCR production [11-16]. The basic system uses a pPIC9K vector (Invitrogen) where GPCR expression is under the control of the strong methanol inducible AOX1 promoter. Protease deficient expression strains, such as the SMD1163 strain, and the use of the α-factor leader sequence have improved receptor expression levels [11,12]. Modifications to the growth media including addition of histidine, receptor specific ligands and dimethyl sulfoxide, which facilitates phospholipid biosynthesis and membrane proliferation in yeast [17] have been shown to increase the expression levels of 20 different GPCRs in *Pichia *[15]

An additional advantage to the use of *Pichia *is that it readily adapts to large-scale culture in bioreactors [18]. Bioreactors allow precise regulation of the aeration, pH and addition of carbon source, which in turn allows the cultures to grow to ultra-high cell densities maximising expression of the target protein. However optimisation of standard protocols is usually necessary for specific targets. One particular issue is the amount of methanol added for induction. Indeed, very high levels of methanol can induce cytotoxic effects which reduce cell viability and thus expression [19]. Methanol sensors, which detect the level of unmetabolised methanol have been key to reducing these cytotoxic effects. Another important issue to consider is the osmotic stress induced during high cell density culturing which is known to be responsible for adaptative cell response mechanisms, such as changes in the membrane lipid content [20]. This may not be desirable in the process of membrane protein production and medium cell density culturing approach may appear more suitable. One aim of this study was to develop a protocol for the large scale expression of GPCRs in *P. pastoris *using bioreactors. The target GPCR used for development and characterisation of the protocol was the human Adenosine A_2A _receptor (A_2A_R), one of four receptors mediating the effects of adenosine. The A_2A_R is key in downregulation of immune responses *in vivo *and this in turn prevents inflammatory damage of healthy tissue [21]. In addition, it has been implicated in several serious conditions including cancer, Parkinsons's disease and asthma. A number of studies have previously shown heterologous expression of this receptor in a range of expression hosts including *Escherichia coli *[22], *Pichia pastoris *[16] and *Saccharomyces cerevisae *[23]. The A_2A_R expressed in *E. coli *was sensitive to C-terminal proteolysis upon solubilisation [22]. Recent studies have shown the importance of removal of the unstructured C-terminal tail, among other modifications, in obtaining well diffracting crystals and ultimately structure determination of GPCRs [24,25]. The A_2A_R has a particularly long C-terminal tail, comprised of approximately 96 amino acid residues, likely to reduce the chances of obtaining well diffracting crystals. Therefore we also wished to assess the effects of generating a C-terminal truncation on the functional expression and stability of the receptor. A receptor construct was generated truncating the protein at valine 334 (V334), a residue corresponding to the end of the rhodopsin C-terminal tail.

Using a previously described vector system [15] for expression we have developed a large-scale fermentation protocol which produces significantly higher levels of functional A_2A_R than the equivalent volume of culture using published shaker flask protocols [15,16]. This study has also identified a C-terminally truncated version of the A_2A_R receptor which is more highly expressed and more stable than the WT. This construct may be more suitable for downstream functional and structural studies.

## Methods

### Materials

Yeast nitrogen base and yeast extract were purchased from Difco, peptone and L-histidine from Sigma-Aldrich and dimethyl sulfoxide (DMSO) from Acros Organics. Complete EDTA-free protease inhibitor cocktail tablets were purchased from Roche and the bicinchoninic acid assay (BCA) kit purchased from Pierce. Scintillation cocktail (Ultima Gold MV) was obtained from PerkinElmer. [^3^H] ZM 241385 was obtained from American Radiolabelled Chemicals Inc. while ZM241385 was obtained from Tocris and Theophylline from Fisher Scientific. Nitrocellulose membrane was obtained from Millipore and GF/B filters were from Whatman. The mouse M2 anti-FLAG antibody was from Sigma-Aldrich and the sheep anti-mouse IgG-horseradish peroxidase (HRP) conjugate from GE Healthcare. All other chemicals were obtained from Sigma-Aldrich.

### Expression constructs

The WT A_2A_R was expressed using a modified pPIC9K (Invitrogen) vector incorporating a Flag tag followed by a 10 His tag and a TEV cleavage site all upstream of the gene coding for the receptor as described by Andre et al, [15]. This vector was further modified to remove the Bio-tag located downsteam of the gene coding for the receptor. The forward primer 5'-GGT GGA TCC ATG CCC ATC ATG GGC TCC TCG GTG TAC-3' together with the reverse primer 5'-CAT GGA ATT CAC TAG TGA CCT GCT CTC CGT CAC TGC CAT GAG CTG CCA AG-3' introducing a stop codon after residue V334 was used to amplify the truncated A_2A_R gene fragment. This amplified gene fragment was sequenced and cloned into the *Bam*HI and *Spe*I restriction sites of the modified pPIC9K (Invitrogen) vector. Expression plasmids were transformed by electroporation into SMD1163 *P. pastoris *cells.

### Small scale cultures

Single *P. pastoris *colonies containing the plasmid of interest were selected on YPD plates [1% (w/v) yeast extract, 2% (w/v) peptone, 2% (w/v) dextrose, 2% (w/v) agar] containing 0.25% geneticin. Cells from a single colony were used to inoculate 5 ml of BMGY medium [100 mM potassium phosphate pH 6.0, 1% (w/v) yeast extract, 2% (w/v) peptone, 1.34% (w/v) yeast nitrogen base without amino acids, 0.00004% (w/v) biotin, 1% (w/v) glycerol]. The culture was grown overnight at 30°C to an OD_600 _of 12–15. The cells were spun down at 3,000 *g *for 5 min and the cell pellet was resuspended in typically 10 ml BMMY [similar to BMGY with the following changes: phosphate buffer at pH 8.0, 2.5% (v/v) dimethyl sulfoxide, 0.04% (w/v) histidine, and 0.5% (v/v) methanol instead of 1% glycerol] to achieve a starting OD_600 _of 5 for all cultures. The culture was incubated for 18 h at 22°C and then the cells harvested by centrifugation at 5000 *g *for 5 mins. The highest expressing clones were selected by Western blot for further study.

### Flask cultures

Single *P. pastoris *colonies from high expressing clone were selected on YPD plates containing 0.25% geneticin. Cells from a single colony were used to inoculate 50 ml of BMGY medium. The culture was grown overnight at 30°C to an OD_600 _of 3–5. A total of 500 ml of BMGY was inoculated with 25 ml of the starter culture and grown for 6–8 h to an OD_600 _of 5–10. The cells were spun down at 3,000 *g *for 20 min and the cell pellet was resuspended in 500 ml BMMY. The culture was incubated for 20 h at 22°C and then the cells harvested by centrifugation. Samples (1 ml) were taken at various time points during expression (0, 4, 14, 16, 18, 20 hours post induction). The cells were harvested and stored at -80°C for further analysis.

### Bioreactor cultures

Single *P. pastoris *colonies containing the plasmid of interest were selected on YPD plates containing 0.25% geneticin. Cells from a single colony were used to inoculate a 150 ml (or 500 ml for inoculation of a 20 L bioreactor vessel) starter culture of MGY medium (0.1 M potassium phosphate pH 6, 1.34% yeast nitrogen base, 0.0004% biotin, 1% glycerol). The culture was grown at 30°C with aeration for 18–22 hours to an OD_600 _~10–15. The entire inoculum was added to a 5 L fermenter vessel at t = 0. Cultures were grown in a 5 L bioreactor vessel controlled by an ADI 1010 bio-controller connected to a PC running BioExpert software (all from Applikon Biotechnology). A methanol sensor (Raven Biotech) was used to monitor and control methanol levels in the vessel. The internal regulation software of the sensor was used to control the external peristaltic pump and adjust methanol concentration to the set point. Agitation was set at 1000 rpm, pH at 5.0 and dO_2 _at 35% for the entire run. For the initial cell growth phase the temperature was set at 30°C. In this initial phase, the culture was allowed to consume all the glycerol contained in the 4 L of FM22 culture medium (4.3% (w/v) monobasic potassium phosphate, 0.5% (w/v) ammonium sulphate, 0.1% (w/v) calcium sulphate, 1.43% (w/v) potassium sulphate, 1.17% (w/v) magnesium sulphate) containing 2% (w/v) glycerol and 1 ml/L PMT4 trace elements solution (0.2% (w/v) copper sulphate, 0.008% (w/v) sodium iodide, 0.3% (w/v) manganese sulphate, 0.02% (w/v) sodium molybdate, 0.002% (w/v) boric acid, 0.05% (w/v) calcium sulphate, 0.05% (w/v) cobalt chloride, 0.7% (w/v) zinc chloride, 2.2% (w/v) iron sulphate, 0.02% (w/v) biotin, 1 ml/L sulphuric acid). During a second growth phase the culture was fed 50% glycerol at a rate of 3.5 ml/L/h until the OD_600 _reached approximately 75 (~3–4 h). The temperature of the vessel was then reduced to 22°C and the cells treated with a 0.1% v/v aliquot of 100% methanol supplemented with 4 ml/L PMT4, 3% DMSO, 0.04% histidine and the methanol sensor signal allowed to stabilise. The signal output was recorded in mV and the methanol feeding system set point was set to this value. The culture was allowed to adapt to 0.1% methanol for 5 h. For the induction stage, the methanol concentration was raised to 0.5% v/v in steps of 0.1% v/v every 2 to 3 hours. At the end of the induction period (~18 h) the cells were harvested by centrifugation at 3,000 *g *for 5 min. Samples (1 ml) were taken at various time points during the culture (6, 4 and 2 hours pre induction and 0, 3, 6, 9, 12, 15, 18, and 21 hours post induction). The cells were harvested and stored at -80°C for further analysis.

### Membrane preparation

Cells were resuspended in ice-cold breaking buffer (50 mM HEPES-NaOH pH 7.4, 100 mM NaCl, 10% (w/v) glycerol, 2 mM EDTA, 1 mM PMSF, 300 nM ZM241385, (1/100 ml) PI), and broken using a tissue lyser (Qiagen) set to 30 MHz for 15 mins. Cell debris, including intact cells, were removed by a low speed spin (3,000 *g*) for 10 min. The supernatant was retained, and membranes isolated by centrifugation at 100,000 *g *for 30 min. Each membrane pellet was re-dissolved in a membrane buffer (50 mM HEPES-NaOH pH 7.4, 100 mM NaCl, 10% (w/v) glycerol) and flash frozen in liquid nitrogen for storage.

### Radioligand binding assays

All radioligand binding assays were performed using the A_2A_R antagonist [^3^H] ZM241385 in binding buffer (20 mM HEPES, 1 mM EDTA, 1 mM EGTA, 0.1% BSA, pH 7.4). Single-point binding assays on yeast membrane-bound receptor contained 2 μg of membrane protein in a final volume of 1 ml in the presence of a saturating concentration (4 nM) of radioligand at 25°C for 3 hours. Non-specific binding was determined in the presence of 10 mM theophylline, an A_2A_R antagonist and for each time-point the assay was performed in triplicate. Following incubation, bound and free radioligands were separated on Whatman GF/B filters using a cell harvester (Brandel). The filters were washed three times with ice-cold phosphate buffered saline, and the amount of bound radiolabel assessed using a LS 6500 scintillation counter (Beckman Coulter). Binding data were analysed by non-linear least squares fitting using the computer package GraphPad Prism.

### Protein concentration

All protein concentrations were determined by Lowry assay using bovine serum albumin as standard [26].

### SDS-PAGE and Western blotting

For Western-blot analysis the proteins were separated on a 4–12% Bis-Tris NuPAGE gel prior to transfer to a PVDF membrane using the X-Cell system (Invitrogen). The blot was probed with 1:10,000 dilution of primary anti-FLAG M2 (Sigma) antibody followed by a 1:10,000 dilution of secondary goat anti-mouse horseradish peroxidase antibody. Protein bands were visualised using ECL substrate (GE Biosciences).

## Results

### Bioreactors allow controlled cell growth

**Figure 1:**
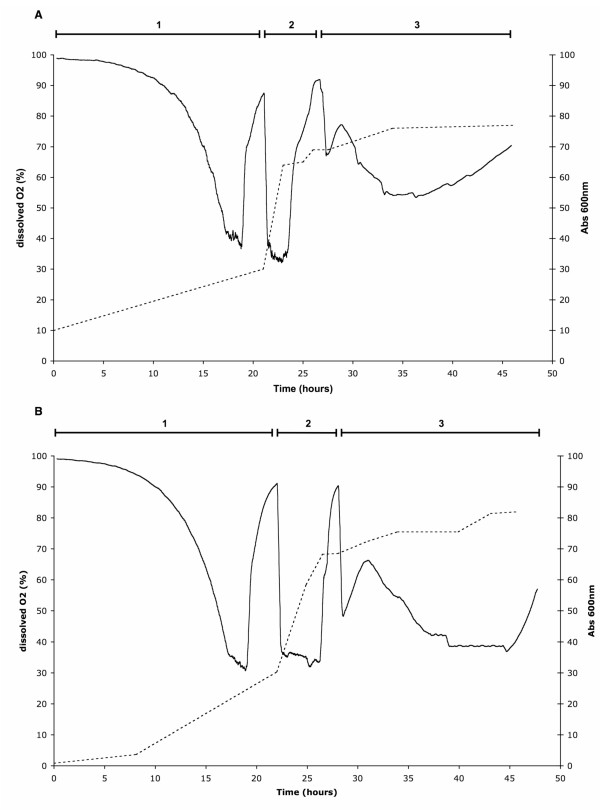
**Comparison of key bioreactor culture parameters**. Changes in OD_600 _(dashed line) and dissolved oxygen (solid line) over time during the bioreactor culture of the (A) WT A_2A_R and (B) V334 A_2A_R are indicated. Time periods indicated by the numbers correspond to (1) glycerol batch phase (2) glycerol fed-batch phase and (3) methanol induction phase.

Figure 1 shows the changes in two parameters: dissolved oxygen (dO_2_) and OD_600 _during a sample bioreactor culture for both WT A_2A_R (A) and V334 A_2A_R (B) constructs. The initial glycerol batch phase (phase 1) showed a gradual decrease in dissolved oxygen (dO_2_) levels followed by a stabilisation at 35%, the configured set point. As the cells multiply and consume glycerol, the culture's requirement for oxygen increased, lowering the observed dO_2 _in the vessel. Once the 35% set point was reached, the system maintained dO_2 _levels by modulating the airflow. This ensured the culture did not starve of oxygen until all the batch glycerol was exhausted. This phase lasted approximately 22 hours with minor differences depending on the OD_600 _of the starting inoculum. A sharp rise in dO_2 _after ~19 h cultivation indicated that the cells were starving for glycerol and did not require oxygen to metabolise the carbon source. The glycerol fed-batch phase (phase 2 on Figure 1) was then initiated. The culture was fed 50% glycerol at 3.5 ml/L/hour until the OD_600 _reached approximately 75. This caused dO_2 _levels to drop and stabilise again at 35%. This phase lasted between 3 and 4 hours. The culture was then allowed to consume any remaining glycerol before the start of the induction phase. Protein expression was induced by the addition of methanol. An adaptation period of 3–5 hours was required during which the cells alter their metabolism to accept methanol as the new carbon source. This was observed as a steady decrease in dO_2 _levels as the cells slowly adapt to methanol utilisation. During this phase the OD_600 _remained relatively stable at about 75 for the WT A_2A_R construct while typically a small increase was observed for the V334 A_2A_R (from 75 to ~80). The highly similar dO_2 _and OD_600 _traces for the two different constructs demonstrate the reproducibility of the bioreactor culture protocol described in this study.

### Western blot analysis of WT A_2A_R and V334 A_2A_R expressed in flask and bioreactor cultures

**Figure 2:**
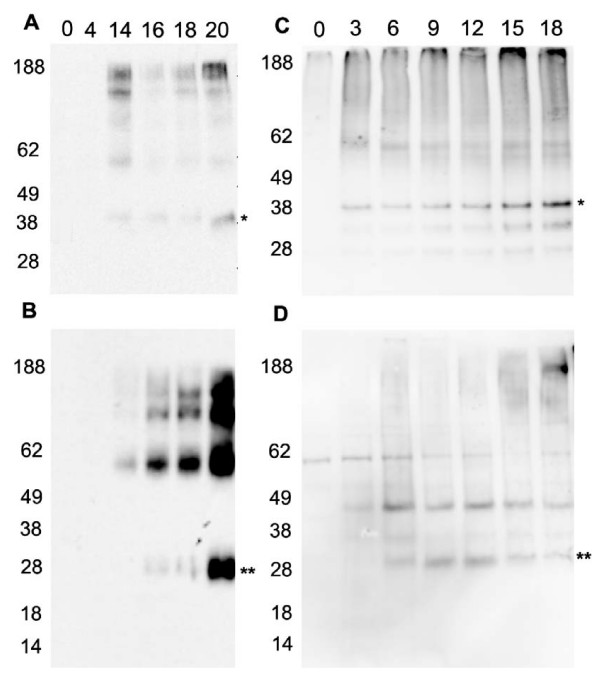
**Expression of the A_2A_R constructs over time**. Western blot analysis of WT A_2A_R (A) and V334 A_2A_R (B) expressed in flask and WT A_2A_R (C) and V334 A_2A_R (D) in bioreactor cultures. Analysis was performed on membranes prepared from cells harvested at the different time points using anti-Flag antibody. M denotes the molecular weight markers and the numbers along the top of blots correspond to the time points.

Expression of the two different receptors during the induction phase for both flask and bioreactor cultures was monitored using both Western blot analysis and radioligand binding assay. Western blot analysis of flask cultures expressing WT A_2A_R showed a gradual increase in expression over the course of the induction phase with receptor protein first detectable at 14 hours post induction (Figure 2A) as a band of approximately 38 kDa. Several larger bands are also visible. These are likely to be due to different oligomeric forms of the receptor formed as a result of gel running conditions rather than representing the true state of the receptor. Due to the large gap in sampling times it is likely that receptor is expressed before 14 hours, however it is clear that no receptor is expressed at 4 hours post induction. The amount of WT A_2A_R did not seem to significantly increase after this. The V334 A_2A_R expressed in flasks (Figure 2B) was first detected as a band of approximately 28 kDa at a similar time point however the amount of protein increased significantly after this to the 20 hour time point where the culture was harvested. Expression of the WT A_2A_R in the bioreactor also increased with time although the expression was detectable by Western blot at 3 hrs in contrast to the flask cultures (Figure 2C). A similar trend is observed for the V334 A_2A_R (Figure 2D). There were some low molecular weight bands visible for the WT A_2A_R, indicating that there is proteolytic degradation of the expressed receptor. The fact that these bands are being detected using the N-terminally positioned Flag tag suggests that the bands observed are due to C-terminal degradation. This is not observed in the flask culture for either construct, or the bioreactor culture expressing the V334 A_2A_R. Interestingly however there is a distinctive band visible at ~45 kDa for the V334 A_2A_R bioreactor samples which is not present in the flask culture for the same construct. It is possible that the higher levels of expression detected for the V334 A_2A_R in the bioreactor stimulates a larger amount of dimer formation in the membrane.

### Functional analysis of WT A_2A_R and V334 A_2A_R expressed in flask and bioreactor cultures

**Figure 3:**
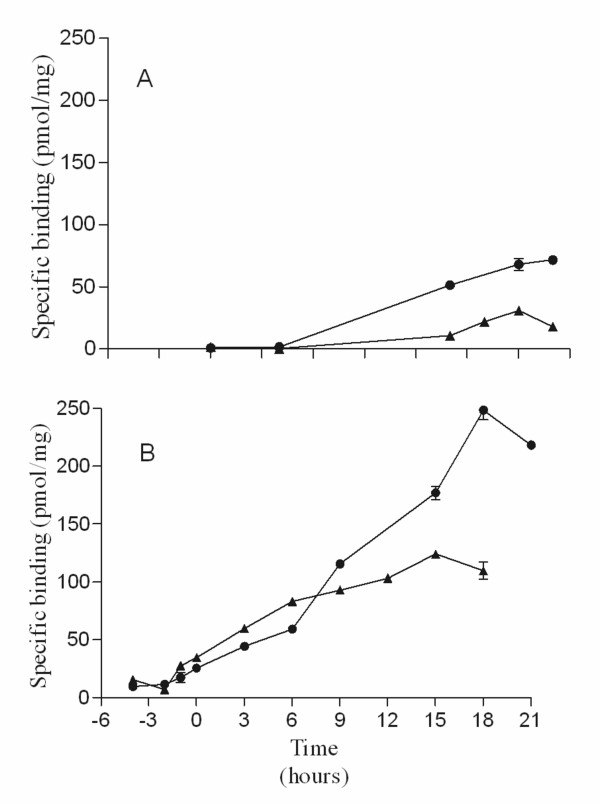
**Functional expression of the A_2A_R constructs over time**. Radioligand binding analysis of both the WT A_2A_R (filled triangles) and V334 A_2A _R (filled circles) receptors expressed in flask (A) and bioreactor (B) cultures. Membranes prepared from cells harvested at the different time points were assessed using radioligand binding assay using [^3^H] ZM241385. Induction of the cultures was initiated by addition of methanol at time 0 hours. Data shown are representative of at least n = 2 experiments for each condition.

Radioligand binding analysis indicated a similar trend in increasing expression of functional receptor from the flask cultures. There was no measurable functional receptor for the first 4 hours post induction (Figure 3A) while after this time functional expression increased rapidly, as measured at 14 hours. In the case of the WT A_2A_R the expression level does not increase much after 14 hours with a final expression level of 23 pmol/mg. The A_2A_R V334 construct expresses to a much higher level after 14 hours (53 pmol/mg) and increases significantly after that to a final expression level of 72 pmol/mg after 20 hour (Figure 3A). The expression profile is slightly different in the case of the bioreactor cultures. Derepression of the methanol promoter due to depletion of the glycerol during the second growth phase, induces expression from the AOX1 promoter resulting in low level but measurable receptor production prior to induction. For both the WT A_2A_R and the V334 receptor constructs there is a steady increase in expression up to 6 hours post induction (Figure 3B). After this time the expression of the WT A_2A_R continues to increase steadily peaking at approximately 125 pmol/mg at the end of the culture (18 hrs) while A_2A_R V334 increases much faster peaking at 250 pmol/mg at 18 hrs (Figure 3B). The culture for the V334 construct was continued for a further 3 hours however the data suggests that this results in a slight drop in functional expression. It may be that 18 hrs is the optimal harvesting time for the bioreactor cultures.

## Discussion

### Flask versus bioreactor expression

The main aim of the study was to compare the expression of A_2A_R in flask and bioreactor based cultures. The flask conditions used in this study were similar to, or the same, as those which have previously been used for expression of the A_2A_R in *P. pastoris *[15,16]. Flask cultures enable the exploration of several expression conditions in parallel and require a limited set-up compared to bioreactor based cultures. However there are a number of drawbacks to flask cultures, which the bioreactor is particularly well suited to overcome. The principal advantage of the bioreactor set up is that it enables large volumes of air to be circulated within the culture to satisfy the high oxygenation requirements of *P. pastoris *cultivation to high OD_600_. The ability to regulate pH and dissolved oxygen (dO_2_) was shown to greatly improve batch-to-batch reproducibility while glycerol and methanol feeding lines enable accurate control over the amount of nutrients added to the culture. *P. pastoris *synthesises alcohol oxidase 1 (AOX1), which metabolises methanol by conversion to formaldehyde. However, if high concentrations of methanol are introduced without pre-conditioning of the culture to this carbon source, excess formaldehyde can accumulate to toxic levels to the cells [27]. It is therefore very important to allow the culture to adapt to methanol utilisation using low methanol concentration. Initial inductions performed in the absence of accurate methanol control systematically produced very erratic expression profiles (data not shown). The implementation of a methanol control system proved essential for the reliability and reproducibility of receptor expression.

The cell density achieved in the bioreactor cultures was routinely almost six times higher than that achieved in flask cultures as assessed by measurement of the OD_600_. In addition radioligand binding indicated that there was an approximately three and a half times higher functional expression for the bioreactor culture compared to the flask culture (summarised in Table 1). Thus the expression level in the bioreactor is increased in two ways; 1) the biomass is increased and 2) the production of functional receptor is increased per mg of membrane protein. These data clearly demonstrate the high efficiency receptor production that can be achieved by the careful regulation and optimisation of the growth parameters possible in bioreactor cultures. It is likely that the protocol described here is suitable for large scale production of other GPCRs in bioreactors since increases in functional expression level have also been obtained for the human dopamine D2 and the human serotonin 5HT1D receptors compared to flask cultures (data not shown).

**Table 1:** Cell densities and specific binding for the A_2A_R constructs expressed in flask and bioreactor cultures

**Construct**	Cell density (OD_600_)*		**Maximum specific binding (pmol/mg)**	
	**Flask**	**Bioreactor**	**Flask**	**Bioreactor**
	
WT A_2A_R	14	75	32.5 ± 9.3	112.5 ± 2.7
A_2A_R V334	14	80	82.9 ± 11.2	200.6 ± 17.6

* cell density at induction

Protein expression in the absence of inducer, so-called leaky expression, is often observed when using recombinant systems [28]. The binding assay results indicated that in the case of both the WT and V334 A_2A_R expressed in bioreactors, receptor was present prior to methanol induction. Glycerol represses the AOX1 promoter used for A_2A_R expression and therefore no receptor should be detected prior to the addition of methanol. However, as the culture is allowed to fully deplete the glycerol present in both glycerol batch and glycerol fed-batch phases, some degree of derepression can occur, allowing some protein to be synthesised. Ligand binding analysis also indicated the presence of small amounts of receptor (maximally ~25 pmol/mg) in bioreactor samples taken prior to induction with methanol. From published data, no more than 2–4% of the total attainable amount of AOX1 induced protein is normally produced through derepression [29]. Interestingly, according to our measurements, an estimated 10–15% receptor was produced in starvation conditions. Functional analysis of the receptor produced as a result of derepression indicates that A_2A_R produced during derepression is correctly folded and functional. The relatively low OD600 values achieved in flask cultures mean it is unlikely that all the glycerol is consumed in the glycerol batch phase explaining why no expression is detectable at induction (time = 0 hours) due to derepression of the AOX1 promoter.

### WT A_2A_R versus V334 A_2A_R functional expression

Radioligand binding analysis indicated that higher levels of receptor were produced for the truncated V334 A_2A_R compared to the WT A_2A_R in both flask and bioreactor cultures with the V334 A_2A_R expressing at approximately double the amount of the WT receptor (Table 1). The B_max _value of 200 pmol/mg achieved for the V334 A_2A_R is the highest reported value for functional expression of the A_2A_R in *P. pastoris*. This is lower than that achieved for expression of the A_2A_R in both *S. cerevisae *(480 pmol/mg; [23]) and mammalian cells (287 pmol/mg; [30]). However the higher cell densities achieved using *P. pastoris *mean that higher expression levels per litre are obtained. Smaller bioreactors are required for *P. pastoris *growth than for S. cerevisae making the cultures both cheaper and easier to handle.

Numerous studies have indicated that C-terminal truncation of GPCRs produces a much more stable protein, more suitable for structural studies [24,25]. Western blot analysis of the WT A_2A_R expressed in the bioreactor indicated the presence of degradation bands likely to be the result of C-terminal degradation. This is a major concern since the His-tag to be used for immobilised metal affinity chromatography is N-terminally located. Thus, purification would not differentiate between intact and degraded receptor. In contrast the V334 A_2A_R does not exhibit this degradation during expression. Previous studies expressing the WT A_2A_R in *E. coli *demonstrated C-terminal degradation upon solubilisation [22]. In addition, work in our group has shown C-terminal degradation of the WT A_2A_R expressed in *P. pastoris *upon purification to high homogeneity (Singh et al, manuscript in preparation). These data indicate an intrinsic instability in the WT A_2A_R. This degradation has not been previously reported for expression of the receptor in relatively small scale *P. pastoris *cultures [16] and this is supported by the findings of this study since no degradation bands were observed for either construct in flask cultures. It may be that the very high expression levels achieved in the bioreactor reduces the processing and targeting time of the receptor making it more vulnerable to degradation by intracellular proteases. The lack of degradation of the V334 A_2A_R indicates that this is more stable and therefore a more suitable candidate for further downstream structural and functional studies. Recent work by Magnani et al, [31] describes the identification of a number of mutants of the human A_2A_R with increased thermostability and increased stability in short chain detergents making them more suitable for crystallisation. Mutant screening was performed in *E. coli*, however it maybe that the use of a eukaryotic system like the large-scale *P. pastoris *system described in this manuscript is more suitable for the production of the mutant receptors for structural studies.

## Conclusion

The use of *P. pastoris *bioreactor cultures has significant advantages over traditional flask cultures for the expression of GPCRs for functional and structural studies. Bioreactors yield higher cell densities as well as higher levels of functional A_2A_R per mg of membrane protein compared to flask cultures. Greater control of both cell growth and induction conditions allow much greater reproducibility of receptor expression. This study has also identified a more stable truncated V334 A_2A_R construct for further functional and structural studies.

## Competing interests

The authors declare that they have no competing interests.

## Authors' contributions

SS and PGS carried out all the radioligand binding assays, SS, AG, CF-V and MM optimised the bioreactor running conditions, AG, CF-V and MM performed the flask cultures for comparison, RR, MM and AG optimised the Western blots, RW and JR made the expression constructs while RW developed the flask expression conditions, BB designed the research and wrote the manuscript.
